# Change in health outcomes for First Nations children with chronic wet cough: rationale and study protocol for a multi-centre implementation science study

**DOI:** 10.1186/s12890-022-02219-0

**Published:** 2022-12-29

**Authors:** Pamela J. Laird, Roz Walker, Gabrielle McCallum, Maree Toombs, Melanie Barwick, Peter Morris, Robyn Aitken, Matthew Cooper, Richard Norman, Bhavini Patel, Gloria Lau, Anne B. Chang, André Schultz

**Affiliations:** 1 Wal-yan Respiratory Research Centre, Telethon Kids Institute, Perth, WA Australia; 2 Perth Children’s Hospital, Perth, WA Australia; 3 Division of Paediatrics, Faculty of Medicine, University of Western Australia, Perth, Australia; 4 School of Indigenous Studies, Poche Centre for Indigenous Health, University of Western Australia, Perth, WA Australia; 5 School of Population Health, University of Western Australia, Perth, WA Australia; 6 Ngangk Yira Institute for Change, Murdoch University, Perth Western, Australia; 7 Child Health Division Menzies School of Health Research, Charles Darwin University, NT Darwin, Australia; 8 Faculty of Medicine, University of Queensland, Brisbane, QLD Australia; 9 Hospital for Sick Children, Toronto, Canada; 10 Department of Psychiatry, Temerty Faculty of Medicine, University of Toronto, Toronto, Canada; 11 College of Medicine and Public Health, Flinders University, Adelaide, SA Australia; 12 Charles Darwin University College of Indigenous Future, Arts & Society, Darwin, NT Australia; 13 School of Population Health, Curtin University, Perth, WA Australia; 14 Northern Territory Department of Health, Darwin, NT Australia; 15 Department of Respiratory Medicine, Queensland Children’s Hospital, Brisbane, QLD Australia; 16 Australian Centre For Health Services Innovation, Qld University of Technology, Brisbane, QLD Australia

**Keywords:** First Nations children, Knowledge Translation, Chronic wet cough, Protracted bacterial bronchitis

## Abstract

**Background:**

In children, chronic wet cough may be a sign of underlying lung disease, including protracted bacterial bronchitis (PBB) and bronchiectasis. Chronic (> 4 weeks in duration) wet cough (without indicators pointing to alternative causes) that responds to antibiotic treatment is diagnostic of PBB. Timely recognition and management of PBB can prevent disease progression to irreversible bronchiectasis with lifelong consequences. However, detection and management require timely health-seeking by carers and effective management by clinicians.

We aim to improve (a) carer health-seeking for chronic wet cough in their child and (b) management of chronic wet cough in children by clinicians. We hypothesise that implementing a culturally integrated program, which is informed by barriers and facilitators identified by carers and health practitioners, will result in improved lung health of First Nations children, and in the future, a reduced the burden of bronchiectasis through the prevention of the progression of protracted bacterial bronchitis to bronchiectasis.

**Methods:**

This study is a multi-centre, pseudorandomised, stepped wedge design. The intervention is the implementation of a program. The program has two components: a knowledge dissemination component and an implementation component. The implementation is adapted to each study site using a combined Aboriginal Participatory Action Research and an Implementation Science approach, guided by the Consolidated Framework of Implementation Research. There are three categories of outcome measures related to (i) health (ii) cost, and (iii) implementation. We will measure health-seeking as the proportion of parents seeking help for their child in a 6-month period before the intervention and the same 6-month period (i.e., the same six calendar months) thereafter. The parent-proxy, Cough-specific Quality of Life (PC-QoL) will be the primary health-related outcome measure.

**Discussion:**

We hypothesise that a tailored intervention at each site will result in improved health-seeking for carers of children with a chronic wet cough and improved clinician management of chronic wet cough. In addition, we expect this will result in improved lung health outcomes for children with a chronic wet cough.

**Trial registration:**

Australian New Zealand Clinical Trials Registry; ACTRN12622000430730, registered 16 March 2022, Retrospectively registered.

**Supplementary Information:**

The online version contains supplementary material available at 10.1186/s12890-022-02219-0.

## Background

In children, a chronic wet cough can be a sign of underlying lung disease such as protracted bacterial bronchitis (PBB) or bronchiectasis [1]. Typically, PBB is diagnosed when a child has a chronic (> 4 weeks duration) wet cough, in the absence of indicators to alternative diagnoses, that responds to antibiotic treatment [2]. Prompt recognition and timely management of PBB can potentially prevent the progression of the disease to irreversible bronchiectasis with lifelong consequences [3]. However, multiple factors often hinder timely recognition and management. For example, a recent study demonstrated if parents considered chronic wet cough normal in their child, medical help-seeking could be delayed [4]. Medical management, in turn, can hinder diagnosis and treatment when there is a lack of disease-specific knowledge, lack of clear and accessible clinical practice guidelines, systems that do not facilitate follow-up of a patient by the same doctor, and other reasons [5].

To recognise and treat PBB appropriately, clinicians must take an accurate medical history, asking in a culturally secure manner about the presence, duration, and quality of cough. They then must exclude pointers to non-PBB related causes of cough such as asthma. Finally, to optimally manage PBB, clinicians must prescribe courses of specific antibiotics of longer duration than they typically would prescribe for many other common conditions [6, 7]. The early detection of PBB typically needs to occur in a primary care setting. Clinicians must be skilled in managing many conditions and practice under time pressure with many competing priorities under working conditions that are not always conducive to detecting and managing conditions that traditionally have been less well known. The challenge is particularly pronounced in children from First Nations, where complex cultural, socio-economic, language, and historical factors interlink with health system factors such as cognitive bias and institutionalised racism [5, 8].

Facilitating the timely detection and management of chronic wet cough and PBB in First Nations children is challenging and requires a comprehensive approach guided by participatory action research and implementation science with an equity, diversity and inclusion lens. Specifically, the timely health care seeking for chronic wet cough by First Nations carers can potentially be facilitated by the provision of specific culturally secure health information through a knowledge dissemination strategy [9]. The timely detection and management of chronic wet cough by clinicians can potentially be facilitated through the implementation of a program at the primary care level [5].

This multi-centre trial will measure the effectiveness of a dual, simultaneous knowledge dissemination strategy for families and implementation component for primary care clinics, adapted for individual regions/communities and their respective primary care clinics, to improve the health of children with a chronic wet cough. We will then employ a health economic analysis to determine the costs and benefits of these strategies to inform future scale-up.

The protocol follows the "The Standard Protocol Items: Recommendations for Intervention Trials [10]" (SPIRIT)10. Reporting will follow the CONSORT Guidelines [11], and, where appropriate, the Standards for Reporting Implementation Research (STaRI) [12]. Reporting of qualitative data will follow the Consolidated Criteria for Reporting Qualitative research [13] (COREQ).

### Overall aim

To facilitate timely detection and optimal management of chronic wet cough in children at primary care clinics.

### Specific objectives

To implement a program that will:

- 1. Improve carer health-seeking for chronic wet cough
- 2. Improve detection and management of chronic wet cough in clinics
- 3. Improve the cough-related quality of life of children who presented to the clinic with a chronic wet cough
- 4. Determine the health-related economics of the program.

## Methods

### Trial design

**Fig. 1:**
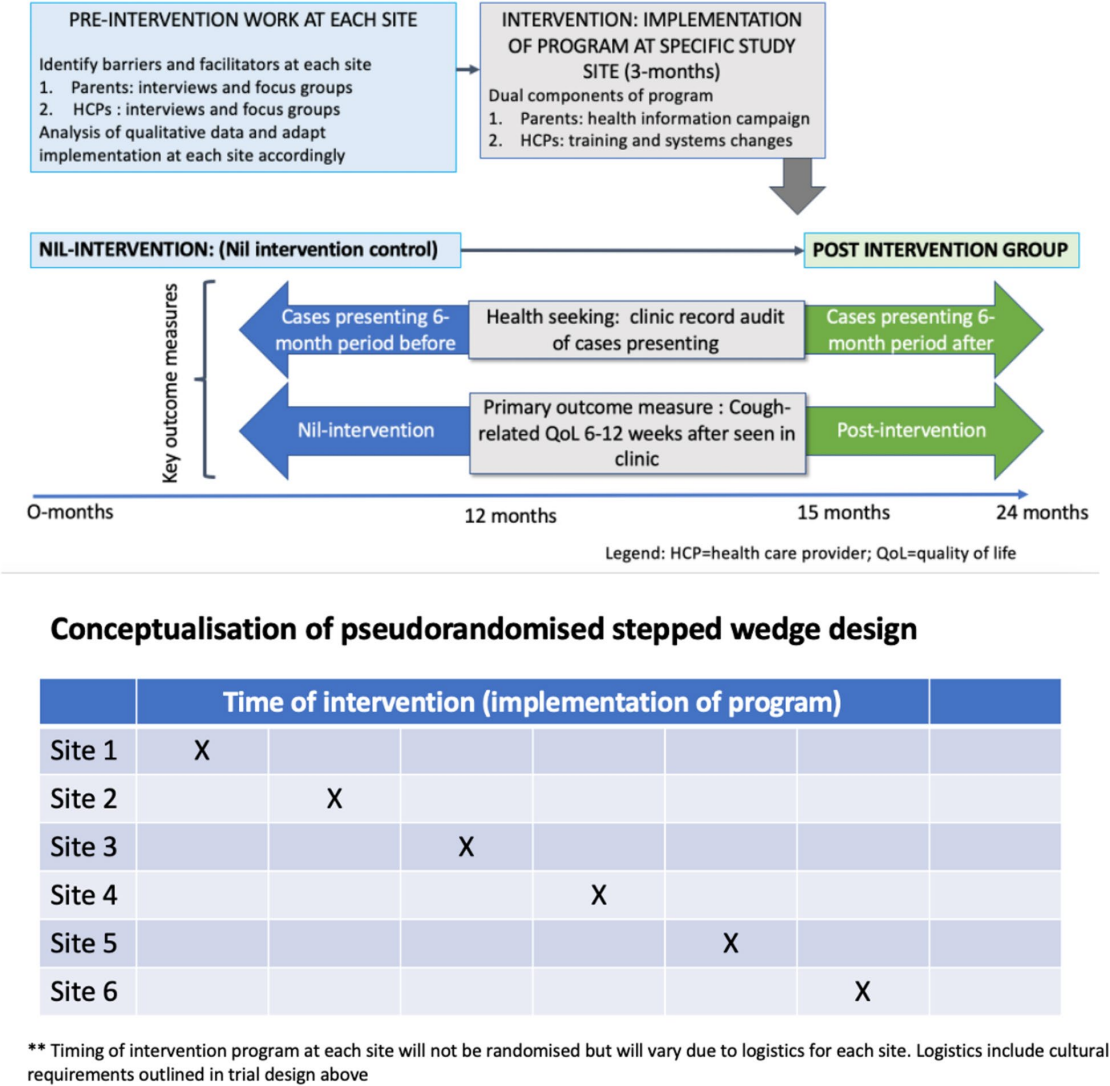
Basic trial structure and design for each study site. ** Legend: HCPs = Health care providers; PC-QoL = Parent Cough related quality of life questionnaire. Timing of intervention program at each site will not be randomised but will vary due to logistics for each site. Logistics include cultural requirements outlined in trial design above

The trial is a multi-centre, pseudorandomised, stepped wedge design. The intervention is the implementation of a program. The program has two parts: a knowledge dissemination strategy for the community and an implementation strategy for the clinics/HCPs, (Fig. 1). The implementation is adapted to each study site using a combined Aboriginal Participatory Action Research and Implementation Science approach [14], informed by the Consolidated Framework of Implementation Research [15]. The program has seven-core components to be implemented and will be tailored to each site (Table 1). Figure 1 provides the trial structure and timing. Pseudo randomisation is a pragmatic approach as the timing of intervention at each site will not be randomised but will vary due to logistics for each site. Logistics include cultural requirements with unpredictable timing, such as lore (custom practices related to coming-of-age) and sorry business (practices associated with the death of a community member), seasonal flooding or other climatic events, and COVID-19 related restrictions to community access.

**Table 1:** Core components of chronic wet cough detection and management program

Core program components	How the component is provided	Who will provide/deliver the component?	Outputs	Outcomes (How the component is delivered as intended)
1. First Nations lead^a^	• Leadership and advice on all cultural components	• First Nations person	• Operations are culturally secure with First Nations knowledge and wisdom integral	• First Nations lead appointed at commencement for duration of study
2. Barrier identification^a^	• Stakeholder engagement (focus groups and interviews) to identify barriers and facilitators to the program • Inner setting: parents and wider community members, HCPs, executive • Outer setting: key stakeholders such as First Nations controlled health organisations, local hospital HCPs, including pediatricians, daycares, playgroups, and a consumer reference group	• Qualitative experts will undertake interviews/focus groups and provide analysis • Interviews and focus groups will gather views of parents, wider community, HCPs	• Identified barriers and facilitators	• Summary of findings, and implementation of program for each site
3. HCP Training (3-month period)	• In-person training/webinar. Series of 3 sessions, 1-month apart • E-learning i.e., 2 online training modules and a podcast(s) accessible and free of charge (will be available in the long term and after the implementation period) • Each clinician to attend at least 3 training sessions, where participation in an e-learning module could replace a face-to-face a training session	• In-person clinical training by pediatric respiratory clinician • Local champion will promote module and podcast at each site	• Module and podcast hosted on website of recognised national body • Clinical/cultural training accomplished	• Metrics of completions at each site (electronic capture system) • Percentage of HCPs employed at each site who received training • Audit and feedback: medical records audited for HCP management of chronic wet cough. Feedback given at monthly intervals
4. Educational resources for HCPs	1. Health information flip chart/leaflets 2. Animated film version of flip chart 3. Flow chart on wall in clinic as reminder	• Champion at each site ensures availability and accessibility of documents	• Educational resources adapted for each site (including language translation) • Available on internet/intranet	• Record whom information resources are given to
5. Practice changes	• Ensure that clinical practice guidelines easily accessible • Call-back option (ability to schedule follow-up of patient by same clinician) • Electronic decision support, if feasible, e.g., prompts to ask about cough quality and duration. Ensuring clinical practice guidelines (a precondition) easily accessible • Provision for nurses to allow initiation of medication treatment context-specific to chronic wet cough • Facilitate medication dispensing, e.g., home visits by health staff, patients attending clinic to receive their medication can bypass triage for medication administration	• Clinical manager to implement practice changes	• Booking system for recall of patients operational • Flow charts placed in clinic rooms • Flexibility in medication dispensing system	• Record if booking option operational • Record flow chart placements • Record of clinic-assisted medication administration • Record of nurses who complete training and can initiate medication treatment
6. Local champions	Facilitated uptake of new processes by local HCPs	• Identified by research team and/or clinical lead at each site	• Champion is trained and at each site	• Presence of champion and input from champion at each site
7. Health promotion campaign (Same 3-month period as HCP training)	1. Health promotion messages on chronic wet cough disseminated at places often attended by members of community e.g. via posters in clinic and community; social media (e.g., Facebook); radio, television 2. Health promotion team-leading community discussions i.e., "yarning" 3. Other information materials and booklets	• Research team to coordinate campaign in liaison with local champions and stakeholders • Health promotion team: respiratory clinician provides training to a central AMS health promotion team (not part of the individual clinics) – trained members and local navigator provide yarning groups. This method of health promotion for regions where social media is limited by slow internet	• Adapted health information materials completed for each site • Health promotion team mobilised to work in community to yarn in small groups	• Check posters placed as required • social media analytics • Number attendees and yarning groups for each community

*Legend*: *HCP* Health care provider, *AMS* Aboriginal Medical Service, *PBB* Protracted bacterial bronchitis

^a^Components 1 and 2 will occur before the 3-month long implementation period

### Participants, interventions, and outcomes

#### Study setting

We will conduct the study in six Aboriginal primary care clinics in three different Australian States and Territories, i.e., Western Australia, Queensland and the Northern Territory and the communities served by the respective clinics.

Sites:

Western Australia:

1. Kimberley regional town (Eastern region): Government and Aboriginal primary care services
2. Kimberley East (3-remote Aboriginal communities)
3. Perth city (Aboriginal primary care service)

Queensland:

1. Central Queensland regional town (Aboriginal primary care service)

Northern Territory sites to be advised.

The trial sponsor is the Child and Adolescent Health Service of Western Australia.

### Eligibility criteria

#### Participants

Participants’ inclusion and exclusion criteria are designated for the various aspects of this study relating to addressing the different outcome measures. Outcome measures encompass measurement of health, economic and implementation outcomes.

### Parents’ health-seeking

- **Inclusion criteria:** Australian First Nations children aged 0–8 years who attend local primary care clinic for medical care during a 6-month period during nil-intervention period and the same calendar 6-month period post-intervention.
- **Exclusion criteria:** Non-First Nations children, First Nations children aged over eight years or who do not attend local clinic.

### Clinician management of chronic wet cough

- **Inclusion criteria:** First Nations children aged 0–17 years who presented to primary care clinic with a chronic wet cough 6-weeks to 3-months prior to recruitment.
- **Exclusion criteria:** Non-First Nations children or children who present outside the study period.

### HCPs knowledge of optimal treatment of children (questionnaire)

- **Inclusion criteria:** HCP who attends a training session at a local medical clinic.
- **Exclusion criteria:** HCP who did not attend a training session or does not work at a medical clinic.

### HCPs knowledge of optimal treatment of children (medical record audit)

- **Inclusion criteria:** Australian First Nations children aged 0–8 years who attend local medical clinic for medical care during a 6-month period during nil-intervention period and the same 6-month period post-intervention for a respiratory-related illness.
- **Exclusion criteria:** Australian First Nations children aged 0–8 years who attend local medical clinic for medical care during a 6-month period nil-intervention period and the same 6-month period post-intervention for a condition other than a respiratory-related illness or non-First Nations children or children aged over eight years.

### Who will take informed consent?

Informed consent via a researcher (First Nations researcher available for all First Nations participants), preceded by a plain language explanation of the research study, will be obtained prior to all face-to-face or telephone data collection methods, including group (yarning i.e., informal conversation that is culturally friendly and recognised by First Nations as meaning to talk about something or receive information [16]) discussions and any one-to-one interviews.

### Waiver of consent for medical record audit information

All relevant ethics committees granted a waiver of consent for medical record audits to measure the number of children seeking help for chronic wet cough in the two-6-month periods, pre-and post-intervention.

### Provisions for consent for collection and use of participant data

The confidentiality of participants will be protected throughout the study by using a unique identifier code. The unique identifier code will be kept on a secured, passcode-protected, encrypted research database at the Telethon Kids Institute and can only be accessed by authorised research team members. De-identified data will be stored in REDCap (Research Electronic Data Capture) software (secured, passcode-protected and encrypted research database) [17, 18].

Study participants can withdraw consent to participate at any time.

### Interventions

#### The explanation for the choice of comparators

Each site will have two periods of data collection (i) nil-intervention period and (ii) post-intervention period (Fig. 1).

#### Intervention description

The intervention consists of a program to improve the detection of chronic wet cough and its subsequent management (henceforth termed the "strategy"). It includes a knowledge dissemination strategy for the local community, i.e., health consumers (First Nations parents and their children) and a component specifically for implementing practise change in medical clinics. A brief description of the intervention is as follows:

#### Knowledge dissemination for the local community

A health information campaign that includes small group ‘yarning’, posters in the clinic and strategic community locations, and health promotion messaging (e.g., radio, television, billboards, social media, information booklets).

The main message for families:

- Seek medical attention for your child’s wet cough early. Wet coughs that last longer than four weeks can lead to more serious illness.

#### Medical clinics

Training of clinicians, educational resources, practice changes, easy to follow updated accessible guidelines, local workflow, and practice changes.

The clear message for HCPs:

- When obtaining medical history, ask about cough quality and duration
- Chronic wet cough should be managed according to best practice guidelines
- Acquiring an accurate lung health history requires culturally secure family engagement

The program has seven-core components and is detailed in Table 1. Staff time and resources spent on each task will be documented throughout the study.

#### Criteria for discontinuing or modifying allocated interventions

Program implementation will use a combined decolonising research approach [14] that includes Participatory Action Research (PAR) [13] and consideration of barriers and facilitators identified with the Consolidated Framework for Implementation Research (CFIR). As well, context-sensitive adjustments of implementation processes that engage with First Nations consumer views to ensure meaningful partnerships, consultation, effectiveness, and sustainability, will be used. Identification of adaptations required to implement program core components will involve interviews and focus groups with stakeholders, including:

1. Parents: Purposive sampling with both criterion and snowball sampling [19] to initially recruit parents of First Nations children with recurrent respiratory infections and/or cough, and then more broadly the wider First Nations parent community. At all interviews with First Nations participants, a First Nations researcher will be present.
2. Healthcare providers (HCP): Recruitment of HCPs at the local primary care clinic who are involved in caring for First Nations children. Snowball sampling will be used to ensure wider relevant stakeholders are consulted.

Inclusion criteria of participants for interviews:

1. Parents or carers of First Nations children in the town/community: The pragmatic definition of parents include grandparents, aunts and uncles, foster parents, or other family members within the kinship system, who take on the responsibility of taking care of a child.
2. HCP: Any HCPs within the local clinic who are involved in the medical care of First Nations children, including but not limited to doctors (paediatricians, service coordinators, Aboriginal health practitioners, primary-care liaison personnel), nurses and clerks.
3. External stakeholders: such as Aboriginal Community Controlled Health Councils, peak bodies, hospital HCPs such as paediatricians, nurses, and physiotherapists.

The interviews and focus groups provide insights to understand local knowledge and perspectives of chronic wet cough and the barriers and facilitators to implementing the program. Researchers with qualitative research skills and content experts (clinician or Aboriginal lead depending on participant) will conduct interviews and focus groups. Interview participants will be recruited during the “nil-intervention” period, prior to the implementation of the program.

Interviews and focus groups will be recorded. The interview guides (see Additional file, Appendix 1 and 2) were developed by two researchers with experience in qualitative methods and a pediatric respiratory physician. The draft questions were developed for the first site and then refined. Note all interviewees will be invited at the time of the interview if they would like to review their interview transcript, to confirm or change their information.

Sample size for interviews and focus groups.

1. Parents: ~ 5–20 individual interviews per site.
2. HCPs: ~ 2–3 focus groups with 6–8 participants and ~ 10 individual interviews per site.

Sample size was estimated taking into account the following: the concept of information power [20] and the aim of the study, specificity of the sample, use of established theory, the quality of dialogue to be anticipated and the analysis strategy. Recruitment will continue until data saturation [21] is achieved.

Data analysis for interviews and focus groups.

For the identification of barriers and facilitators to the adaption of the implementation of the program, deductive thematic analysis by applying CFIR constructs will be performed. Implementation factors identified will inform implementation processes at each site. Trustworthiness will be facilitated through addressing the four key criteria outlined by Nowell et. al [22].

### Strategies to improve adherence to interventions

The implementation process, and fidelity at each site will be managed through a tracking log of outputs and with field notes.

### Outcomes

The study outcomes consist of four categories: (i) health-seeking, (ii) health, (iii) economics and (iv) implementation.

i) **Health-seeking**
  This outcome is the number of parents seeking help at the local clinic for their child's chronic wet cough during a six-month period pre-and post-intervention. We will use clinic record audit to compare patients in a 6-month period the year before implementing the intervention, up to patients during 6-months directly after the intervention is implemented. Hypothesis: significantly more parents will seek medical attention for children with ongoing wet cough in the year after the intervention than in the preceding year. Numbers will be normalised to clinic activity over each corresponding 6-month period. To minimise the effect of seasonal factors influencing results, the same six calendar months will be audited pre-and post-intervention and will take into account the seasonal respiratory viral infections.
ii) **Health: Cough-specific Quality of Life (PC-QoL)**
  The primary outcome will be the parent-proxy Cough-specific Quality of Life [23] (PC-QoL) (8-question version) for children with chronic wet cough. Effective management of children with chronic wet cough by clinicians will be assessed by measuring PC-QoL between 6-weeks to 3-months after presenting to the local clinic. The PC-QOL tool is a valid, reliable, and responsive tool for measuring the burden of chronic cough in children [23] and has been used effectively in First Nations settings [9].
  Rationale: Children with chronic wet cough in the absence of alternative causes are likely to have protracted bacterial bronchitis that should resolve if managed with an appropriate antibiotic. Hence, if children receive an appropriate antibiotic, then we expect they will have improved quality of life after 6-weeks to 3-months compared to those who did not.
  The PC-QoL scores will be compared nil- and post-intervention in different children who presented to the clinic with a chronic wet cough. A value of 0.9 is the calculated minimum important difference in PC-QOL to demonstrate a clinically significant improvement [23].

### Secondary outcome measures

The proficiency of clinicians in assessing and managing children presenting with respiratory illness will be assessed via medical record audit of clinicians recording: (i) cough presence, (ii) cough quality, (iii) cough duration, and (iv) correct management if suspected PBB.

The medical record audit period will occur in the same two, six-month periods pre-and post-intervention

iii) **Economic evaluation**
  Economic evaluation will be conducted from a society and health system perspective. Cost data for the economic evaluation will be sourced directly from medical clinic records and will include all costs associated with the intervention, including the training workshops and health information campaigns. We will also collect health care utilisation data 6-months pre-and post-intervention from patient electronic health records. Outcomes for our economic evaluation will be based on the proportion of clinicians adhering to guideline recommendations, as well as patient-relevant outcomes, i.e., the proportion of patients at risk of developing bronchiectasis. Given the non-randomised study design, incremental outcomes for the economic evaluation will be calculated as the difference between proportions before and after the intervention, which adjust for seasonality. The trial-based cost-effectiveness analysis will calculate (a) the cost per additional clinician (compared to pre-intervention) providing optimal management of chronic wet cough and (b) the cost per additional patient (compared to pre-intervention) avoiding bronchiectasis.
  In addition to the above analyses, we will conduct a modelled cost-effectiveness analysis to estimate the cost per case of bronchiectasis prevented, based upon the assumption that at least 40% of children with chronic wet cough have PBB and will develop bronchiectasis if undetected and/or sub-optimally managed. The results will be plotted on a cost-effectiveness plane. Bootstrapping and Monte Carlo simulation will be used to estimate a confidence interval around costs and health outcomes. A one-way sensitivity analysis will be conducted around the key variables. A probabilistic sensitivity analysis will be conducted to estimate the joint uncertainty in all parameters. A cost-effectiveness acceptability curve will be plotted. Such a curve provides information about the probability that an intervention is cost-effective, given a decision maker’s willingness to pay for each additional child avoiding bronchiectasis.
iv) **Implementation outcomes**

Implementation outcomes will be used to measure how successful the program was implemented [24].

- Fidelity to core components of the program will be assessed as per Table 1 outcomes.
- Acceptability for both HCPs and parents (i.e., the perception among implementation stakeholders that the program is agreeable, and satisfactory27) and feasibility (i.e., the practicability and suitability of the program for everyday use 27) will be assessed through semi-structured interviews. (See Additional file, Appendix 1 and 2).
- Appropriateness: (“the perceived fit of the program for the given practice setting”.27) Assessment will be through two tools. Firstly, a 22-item Workshop Evaluation (WEVAL) and secondly a 14-item Workshop Assessment Follow-Up (WAFU). The WEVAL evaluates perceptions of the program directly after implementation ( http://ibr.tcu.edu/wp-content/uploads/2013/06/weval.pdf ) and the WAFU evaluates changes 6-months later. ( http://ibr.tcu.edu/wp-content/uploads/2013/06/wafu.pdf ). (Note, the evaluations have been amended with approval from the authors for local use, e.g., removal of social security number)
- Assessment of implementation cost is described under the economic analysis section below.
- Penetration and adoption (i.e., the degree of update and spread of implementation27) will be assessed as 1) the number of HCPs undertaking training out of the total number of HCPs employed, and 2) audit of medical records per secondary outcome measure description.
- Sustainability (i.e., “the durability of the implementation and the degree it can be maintained”27) will be assessed by questionnaires, interviews and/or focus groups with HCPs and parents in the final months of the study.

The evaluation of implementation outcomes will regard the diversity within First Nations contexts [25]. Assessment of acceptability (b, above) and appropriateness (c above), will include identification of co-design, leadership, governance, and involvement in all processes of implementation by First Nations.

### Participant timeline

The timeline for the study and participants is outlined in Table 2.

**Table 2:**
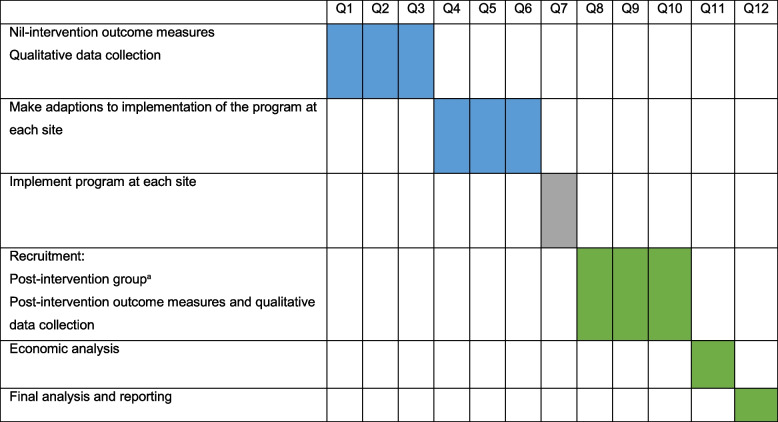
Conceptual timeline of study for ALL sites^a^

*Legend*: *Q* Quarter

^a^The above timeline applies to individual study sites/clusters. The timing of Q1 at each site or cluster will vary

### Sample size

The 8-item version of the PC-QOL will be the primary outcome measure. Unpublished data from our previous research suggests a conservative standard deviation of 1.3, a nil-intervention mean score of ~ 2.7 and a post-intervention mean score of ~ 4.3 The study’s power was examine using two approaches, a ‘per site’ intervention perspective (using a two-sided T-Test methodology) and a ‘whole of study’ intervention perspective (using clustered analysis to control for the within-site variation).

The ‘per site’ perspective suggests that with a minimum of 12 participants (that have chronic wet cough in each period) there will be > 80% power to detect a difference of 1.6 between the nil-intervention and post-intervention periods, alpha 0.05. The ‘whole of study’ perspective suggests that with a minimum of 5 participants per cluster (4) per period there will be > 95% power to detect a difference of 1.6 between the nil-intervention and post-intervention periods (assuming COV 0.65, ICC 0.01, alpha 0.05); with a minimum of 10 participants per cluster (4) per period there will be > 80% power to detect a smaller difference of 0.9 between periods (assuming COV 0.65, ICC 0.01, alpha 0.05). The presented calculations, carried out using PASS [26], are not adjusted for multiple comparisons as they were simply carried out to provide guidance during study design given, ultimately, the achievable final sample size is contingent on both cohort sizes and CWC incidence during the study periods.

### Health-seeking

Our recent study at a single AMS showed that only 1.6% of the 634 children who attended clinic over a 6-month period pre-intervention were seeking help for chronic wet cough. Assuming that health-seeking rates will be similar across study sites, this study aims to at least double this rate at each study site, for which we will have at least 80% power to detect such an improvement at the 0.05 level of significance.

#### Pragmatic data collection

Ultimately, the power of the study will depend on the ability to recruit participants from the limited pool of diagnosed patients within the study population. We estimate that within each of the two stages, somewhere between 12–20 eligible children will be recruited (based on data from the first and completed study site [9]). Thus, we aim to have data for 12 participants per combined regional sites (study cluster) per stage.

### Recruitment

#### Recruitment for QOL survey in nil-intervention and post-intervention groups

**Fig. 2:**
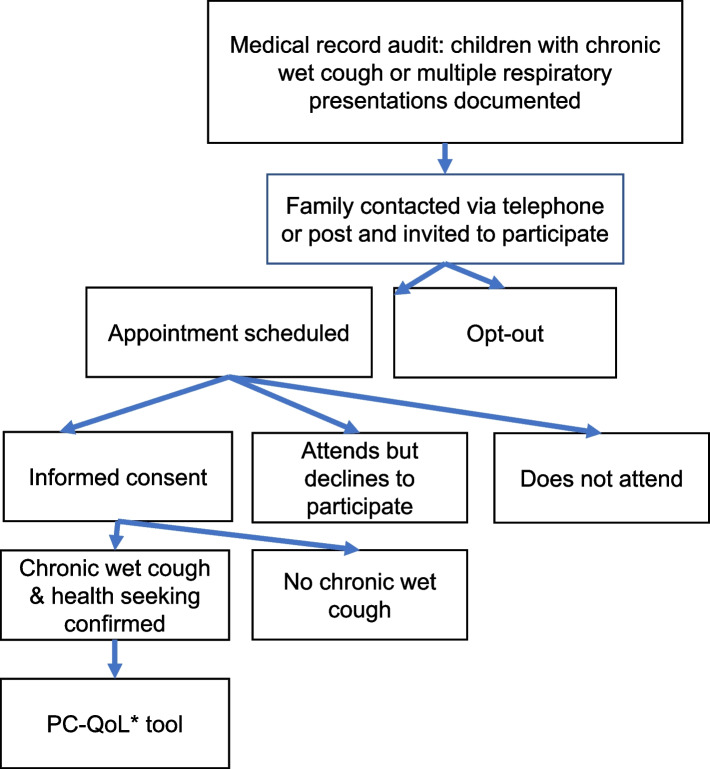
Recruitment process for cough related quality of life surveys. Legend: PCH-QoL = parent proxy cough related quality of life

Participants will be recruited following a medical record audit identifying children who presented for chronic wet cough or who had multiple presentations for cough and chronic wet cough. This will be subsequently confirmed through parental history from interviews conducted by two researchers (including at least one specialist clinician). Children will be excluded if they are non-First Nations, if they do not have a chronic wet cough (i.e., a daily wet cough ≥ four-weeks) or if the family is unable or unwilling to give informed consent (Fig. 2).

#### Recruitment of interviews with parents/carers

Posters advertising the study will be placed in clinic waiting rooms and strategic community settings. Parents who respond to the poster by contacting the study team will potentially be recruited. Parents of children who have presented to the clinic with their child with repeated respiratory infections or for chronic wet cough who are identified for the PC-QoL tool will also be invited to participate in an interview at the time of their appointment for the PC-QoL tool. Snowball sampling will be employed to recruit more widely to ensure views are gathered from other families who do not necessarily have children with chronic wet cough to ascertain knowledge of other community members in general.

#### Recruitment of HCPs

Clinic staff will be identified via the management of the clinic. Hospital staff will be identified via clinical and management leaders. Snowball sampling will be employed to recruit more widely to ensure views are gathered from all key stakeholders in the outer setting.

#### Plans for assessment and collection of outcomes

Health-seeking data will be collected through an audit of medical records by a clinician-researcher and entered into REDCap [17, 18]. Clinician management of chronic wet cough will be collected from medical records, with data entered into REDCap. The PC-QoL questionnaire [23] will be administered to eligible participants. The PC-QoL questionnaire [23] has eight questions rated on a Likert scale, and is administered using REDCap. Qualitative data will be collected via the interviews and focus groups, transcribed, and then analysed, as described in Sect. 11b, page 14.

Site coordinators will regularly monitor data collection and source documents. The study database will be monitored for missing data or errors in data entry.

### Data management

Study data will be stored in REDCap (https://www.project-redcap.org/), using the in-built quality control functionality including data range and consistency checks.

### Confidentiality

All data will be collected with informed consent except where a waiver has been provided by the relevant Ethics Committees. All written materials will be securely stored in a locked office with authorised access only. Data will be stored for at least 15 years as per the National Health Medical Research Council (NHMRC) Guidelines [27].

### Statistical methods

The study’s data will be stored and managed in REDCap, data will be exported for analysis. A range of descriptive statistics will be presented (means and standard deviations; medians and interquartile ranges; counts and percentages) for each site individual and for the full sample, alongside figures if appropriate, which provides a basic overview of the study data. Analysis to address the study’s primary research question will utilise a mixed model framework; here, the outcome will be the absolute PC-QoL score, and the difference between the “nil-intervention” and the “post-intervention” groups will be determined with a 2-level fixed term in the model. The coefficient for this term (and 95% confidence interval) from the model will be reported, as this will represent a ‘whole of program’ effect. The within-site clustering will be controlled for, in the model, by including both a fixed and random term for the study site; if individual participants present during more than one observation periods, this will also be controlled for using a random term in the model. Numerous covariates have been considered for in the modelling, those that will be included (and their justification) are as follows: age, sex and calendar week (to control for risk differences by age, sex and time/seasonal effects respectively). It has been demonstrated that including covariates, such as those listed, typically increases study power [28]. The appropriateness of the model, and the validity of the relevant assumptions, will be assessed using both model fit statistics and through the examination of residual plots. If the final selected model deviates from that described above, justification to support the change will be provided in the report.

Further to the primary analysis outlined above, both a minimally adjusted model (following a similar modelling framework and structure, but with no covariates included the model) and a completely unadjusted model (a Student’s t-test) will be reported. Secondary outcomes (for example, cough presence, quality and duration and management) will initially be assessed using basic comparisons, for example, Chi-squared tests and Student’s t-tests. A mixed model framework (as used in the primary analysis) will be used to quantify the effect of the intervention on prognostic factors of interest (logistic regression for binary outcomes, Poisson regression for count outcomes; with 95% confidence intervals reported alongside effect estimates). The focus of all analysis will be on reporting the effect size estimates (with confidence intervals), as opposed to focusing on statistical significance.

### Interim analyses

No interim analysis is planned.

### Methods in analysis to handle non-adherence to the protocol and statistical methods to handle missing data

Data audits will be completed to query missing entries. Unvalidated data will be excluded from analyses. The study team will regularly verify information that has been collected and monitor for missing data or errors in data entry.

### Oversight and monitoring

#### Steering committee

The APPLE Study Steering Committee, comprised of the Coordinating Principal Investigator, Principal Investigators, and invited collaborators and stakeholders will assume responsibility for the APPLE study design and implementation.

#### Executive committee

The APPLE Study Executive Committee will meet regularly to oversee the day-to-day management of the APPLE study. It will be comprised of key study members and report to the Steering Committee. Day-to-day decisions will be made on minor issues without the need for prior approval. The Executive Committee will ensure decisions made by the Steering Committee are communicated to the Operations Group.

### Operations group

The APPLE Operations Group, comprising the Coordinating Principal Investigator, Study Manager, and selected study staff, will operationalize decisions made by the Steering Committee. The Operations Group will also have oversight of study procedures.

### Consumer reference group

A consumer reference group will advise on study process and receive updates on study progress. The group will include parents and family member whose First Nations child/children have lived experience of chronic lung disease.

### Plans for communicating important protocol amendments to relevant parties (e.g., ethical committees)

Protocol amendments will be documented and submitted to the relevant research ethics and governance committees.

### Dissemination plans

Results of this study will be published and presented in a variety of formats, including presentations at relevant medical conferences and published in peer-reviewed journals. A final report will be disseminated at the conclusion of the study to participating families, consumer reference groups, participating medical clinics as well as to local councils and peak bodies.

## Discussion

Despite massive investment by government, the health gap between First Nations and other Australians is still large and not closing rapidly [29]. Many diverse health conditions contribute to the health gap [30] and only by addressing each of these conditions and the underlying contributing factors will the gap be closed. Respiratory disease contributes significantly to the health gap and PBB, if left untreated, can progress to chronic irreversible and life-limiting respiratory disease, is highly prevalent in children in some First Nation populations [31, 32]. Approaches to address health problems in First Nations have traditionally focused on communities and on health systems separately. We propose an approach that targets families and health systems simultaneously. Our previous research has shown that families are often not aware that chronic wet cough in children is a symptom that requires medical attention [8, 9]. Concurrently, help-seeking by families is futile if the attending clinician is not proficient in managing the condition requiring treatment. As PBB was only formally recognised in 2006 [33], clinicians are often still uninformed about correct screening and management [5]. We hypothesise that a tailored intervention, based on identified barriers and facilitators identified through engagement with carers and health service providers, will result in improved health-seeking by carers of children with chronic wet cough and better management of chronic wet cough by clinicians. We expect this to result in improve lung health children seeking help for chronic wet cough.

### Rationale for our chosen approach

We aim to simultaneously 1) change the help-seeking of parents and 2) clinician practice to 3) improve the health of children with PBB. Initiatives by external agencies in First Nations settings have traditionally been hampered by the lack of involvement of First Nations people [34]. In contrast, our study is a partnership with local First Nations communities. PAR approaches, which have been demonstrated to be effective in First Nation settings [35] will be used in the study. The PAR approach facilitates community empowerment and translation of research into action.

To influence clinician practice, we will use an implementation science approach grounded in the CFIR. The CFIR is among the most comprehensive and widely used frameworks in implementation science [36], which considers a wide range of determinants that have been found to influence implementation outcomes. These determinants will be considered when adapting and individualising the program to site-specific contexts.

A pseudo-randomised stepped wedge trial will be used, with no formal randomisation, because we have already commenced this study, and timing of the intervention will vary significantly between study sites/clusters due to the considerable number of hurdles that must be overcome at each study site before the study can commence. Such hurdles include challenges with accessing remote communities where access can be impossible for extended periods due to flooding, COVID-19 related restrictions to entry by non-essential workers imposed by government, sorry business, local First Nations lore-time of which the timing varies from year-to-year and that can be imposed at short notice. Also, once the need to facilitate appropriate medical help-seeking and optimal medical management of chronic wet cough and PBB becomes clear to local stakeholders, a randomised design becomes unacceptable. The stepwise analysis of change resulting from implementation of the program will, however, provide evidence of the efficacy of the program. Replication at multiple sites will potentially further strengthen the study findings and provide an opportunity for analysis of the relative efficacy of the study intervention at different study sites.

The implementation of the program will be driven by the research team. Our study will determine if the combined PAR-implementation science approach, directed at both families and health systems, is effective across different settings. The study will also determine how the strategy should be adapted to different settings and the cost-effectiveness of our approach. If effective, the team will continue to support the program scale-up in other settings in a purveyor function, and sustainability will be supported by e-module training. The current study will provide ideas on how to automate the identification of site-specific barriers that can be addressed to improve the delivery of the program.

### Trial status

The trial has been approved by the Western Australian Aboriginal Health Ethics Committee on 26.06.2017; approved by the Child and Adolescent Health Service Ethics Committee on 28.07.2020; approved by Human Research Ethics Committee of the Northern Territory Department of Health and Menzies School of Health Research on 23.03.2021; approved by University of Queensland's Human Research Ethics Committee on 09.11.2020. Recruitment has commenced.

## Supplementary Information

**Additional file 1: Appendix 1.** PARENT semi-structured interview guide.

**Additional file 2: Appendix 2.** Health care providers semi-structured interview guide.

**Additional file 3. **

## Data Availability

The datasets used and/or analysed during the current study available from the corresponding author on reasonable request.

## Abbreviations

PBB: Protracted bacterial bronchitis
STaRI: Standards for Reporting Implementation Research
COREQ: The Consolidated Criteria for Reporting Qualitative research [14]
NHMRC: National Health Medical Research Council
PC-QoL: Parent proxy Cough-related Quality of Life
HCP: Health Care Provider
CFIR: Consolidate Framework for Implementation Research
REDCap: Research Electronic Data Capture
