# Supplementary material for: Change in health outcomes for First Nations children with chronic wet cough: rationale and study protocol for a multi-centre implementation science study
Source: BMC Pulm Med. 2022 Dec 29;22:492. doi: 10.1186/s12890-022-02219-0 (PMC9798941; doi:10.1186/s12890-022-02219-0)
Supplement: Supplementary file 1 — Additional file 1: Appendix 1. PARENT semi-structured interview guide. [file 12890_2022_2219_MOESM1_ESM.docx]

**Appendix 1: PARENT semi-structured interview guide**

Characteristics of the individual

Knowledge and familiarity with chronic wet cough:

1. Has your child ever had cold sick or hot sick with a cough?
   1. If yes, can you tell me a bit about what that was like?
2. Have you ever noticed a cough that just doesn’t go away?
3. Has your bubs/child ever had a cough that sounds like there is spit/phlegm that goes on for a long time, but they don’t have a fever, or trouble breathing?
   1. If yes, did you go the doctor or the clinic?
   2. If no, when would you see a doctor for your child’s cough?
      1. If yes, when else would you go to the doctor for your child’s cough?
4. Do you know about (or has someone (a nurse or doctor) talked to you about wet and dry cough?
5. Have you ever heard about wet cough and how lung damage might happen if the wet cough goes on for a long time?
6. Have you ever taken your children to the doctor or hospital with wet cough?
7. Have you ever heard or been told how long a cough could go on for before you should see a doctor?
8. Has any clinic staff ever talked to you about lung health before?

Self-Efficacy:

1. Would you feel confident to take your child to the doctor if they had a wet cough for a long time without a fever or other symptoms?

Intervention characteristics

1. What do you think will be a good way to raise awareness about chronic wet cough?
   1. If interviewee brings up any of the below resources, discuss their ideas and then show example of existing resources.
      1. Poster
      2. Animated film
      3. Radio ad
      4. TV ad
      5. Flipchart
   2. If interviewee does not bring up any of above-named resources, interviewer to ask at the end for interviewee opinion on the existing resources. Ask specifically for any barriers and facilitators to implementing the resources and if any adaption is required. to ask regarding any adaption of resources:
      1. Colour
      2. Wording
      3. Language
      4. Pictures used
      5. Local culture
   3. Which media are most necessary or used in region? (e.g., Facebook, YouTube, Radio, Television, posters or other forms like small group yarning?

Implementation process

1. Who are key people you think we should talk to?
2. Who are key people you think would want to be involved with the implementation?
3. If we rolled out a health information campaign, what would we need to consider locally to do this?
   1. Is yarning important way to promote?
   2. If yarning, who are the influential people in community, who can help promote the health messages?

Inner Setting

Structural characteristics:

1. Have you ever had any problems getting help at the clinic for your child if they had ongoing wet cough?
2. What would help you or make it easier for you to see a doctor for your child’s cough?
3. What would make it easier to give your child medication?

Culture:

1. What is your experience at the local clinic?
2. What is the culture like at the local clinic? Why?
   1. If any issues - How can the culture be more favourable?

Implementation climate:

1. Do you think anything needs to be changed regarding chronic wet cough? If yes, how strongly do you think things need to be changed?
2. How important is this to you that we raise awareness about chronic wet cough?
