# Supplementary material for: Change in health outcomes for First Nations children with chronic wet cough: rationale and study protocol for a multi-centre implementation science study
Source: BMC Pulm Med. 2022 Dec 29;22:492. doi: 10.1186/s12890-022-02219-0 (PMC9798941; doi:10.1186/s12890-022-02219-0)
Supplement: Supplementary file 2 — Additional file 2: Appendix 2. Health care providers semi-structured interview guide. [file 12890_2022_2219_MOESM2_ESM.docx]

**Appendix 2- Semi-structured interview guide health care providers**

Blurb: Explain to clinicians the basics of the intervention:

Aim of interview to ascertain attitude to intervention and barriers and facilitators to intervention being implemented.

Characteristics of the individual

Knowledge and familiarity with chronic wet cough:

1. What do you know about wet cough in children?
2. When would you want to assess or treat a child who has ongoing wet cough?
3. Do you know the length of time an isolated ongoing wet cough goes for before it warrants management?
4. Do you see children with ongoing wet cough i.e., wet cough >1 month?
   1. How often?
   2. How would you manage a child with chronic wet cough?
   3. When would you want to follow up or refer a child with ongoing wet cough?
5. Are you aware of any guidelines to treat ongoing wet cough in children?
   1. What guidelines are they?
   2. Are the guidelines freely available?
   3. How useful are they?
   4. Would you change anything in them?
   5. How often do you use them?
   6. What enablers may assist in use of protocol – e.g., formal training, use of flow charts, hard copies versus electronic?
6. When would you prescribe antibiotics for cough?
7. Would you ever prescribe long, e.g., 2-4-week courses of antibiotics for cough?
   1. When?
   2. If no, why not?
8. What are the main enablers to diagnosing and treating children with ongoing wet cough? That is - what sorts of things have you found helpful, or do you think would help?
9. Do you know if First Nations children admitted to hospital for a chest infection are at risk for further lung damage?

Self-Efficacy:

1. How confident are you when differentiating between the different causes of wet cough?
2. How confident are you when managing ongoing wet cough?
3. Are you able to confidently determine cough quality?
   1. Wet versus dry, versus tight? Etc

Individual identification with organisation

1. What do you think of the local clinic? (interviewee’s perception of the clinic)
2. What are the values of health care providers towards their work?

Intervention Characteristics

1. Explain intervention (see above summary) Do you think the intervention will be suited to clinicians at your clinic?
   1. What specific barriers to implementing the intervention?
   2. What facilitators may help with implementing intervention (teaching component)?
   3. Any adaption required?
      1. Teaching (face-to-face or virtual)
      2. Online education module
      3. Podcast
2. Do you think you will be able to use the education resources (flip chart and film) to raise awareness about chronic wet cough with families? How easy/hard to you think it will be?
3. Do you think you will be able to receive the teaching about chronic wet cough? Is there a time allocated for teaching?

Inner Setting

Structural characteristics:

1. What are the main barriers to diagnosing or managing children with ongoing wet cough?
   1. Patient barriers
   2. Clinician barriers
   3. System barriers
2. Explain system changes component of intervention
   1. What are the barriers to implementing this at your clinic?
   2. What are the facilitators to implementing this at your clinic?
      1. Is there a system that facilitates follow up of children with chronic wet cough by the same clinician? If not, what changes could be put in place?
      2. Is there a system that facilitates medication dispensing to children? If not, what changes could be put in place?
      3. Is there a system that allows health care providers to initiate medication for chronic wet cough if opportunistically identified? i.e., can nurses initiate treatment?
3. If a First Nations child is admitted to hospital for a chest infection – would you be notified?
   1. Is there any routine follow-up?

Culture:

1. What is the work culture like at the local clinic? Why do you think that?
   1. If any issues - How can the culture be more favourable?
2. What is the attitude of health care providers towards chronic wet cough?
3. What is the local clinic like in terms of willingness to change?
4. What is the cultural security like at the local clinic? Why do you think that?
   1. If any issues – how can the cultural security be improved?
   2. Does the clinic have cultural training for its employees? If yes can you expand?

Implementation climate:

1. Do you think anything needs to be changed regarding chronic wet cough? If yes, how strongly do you think things need to be changed?
2. How important is this to you that we raise awareness about chronic wet cough and treat it properly?
3. How does the proposed implementation fit with your own values?
4. How does the proposed implementation fit in with the local clinic?

Readiness for Implementation:

1. What are the staffing levels and turnover like at the local clinic?
   1. If low staffing levels or high turnover – why do you think that is the case?
2. Do you think there will be time for this implementation (for teaching, raising awareness etc)? Why/why not?

Outer setting

1. Is your local clinic linked into other clinics? What is the network?
2. Do you know what health care providers outside your local clinic think about chronic wet cough? How important is it that they are on board with the implementation?
