# Supplementary figures and images for: Change in health outcomes for First Nations children with chronic wet cough: rationale and study protocol for a multi-centre implementation science study
Source: BMC Pulm Med. 2022 Dec 29;22:492. doi: 10.1186/s12890-022-02219-0 (PMC9798941; doi:10.1186/s12890-022-02219-0)

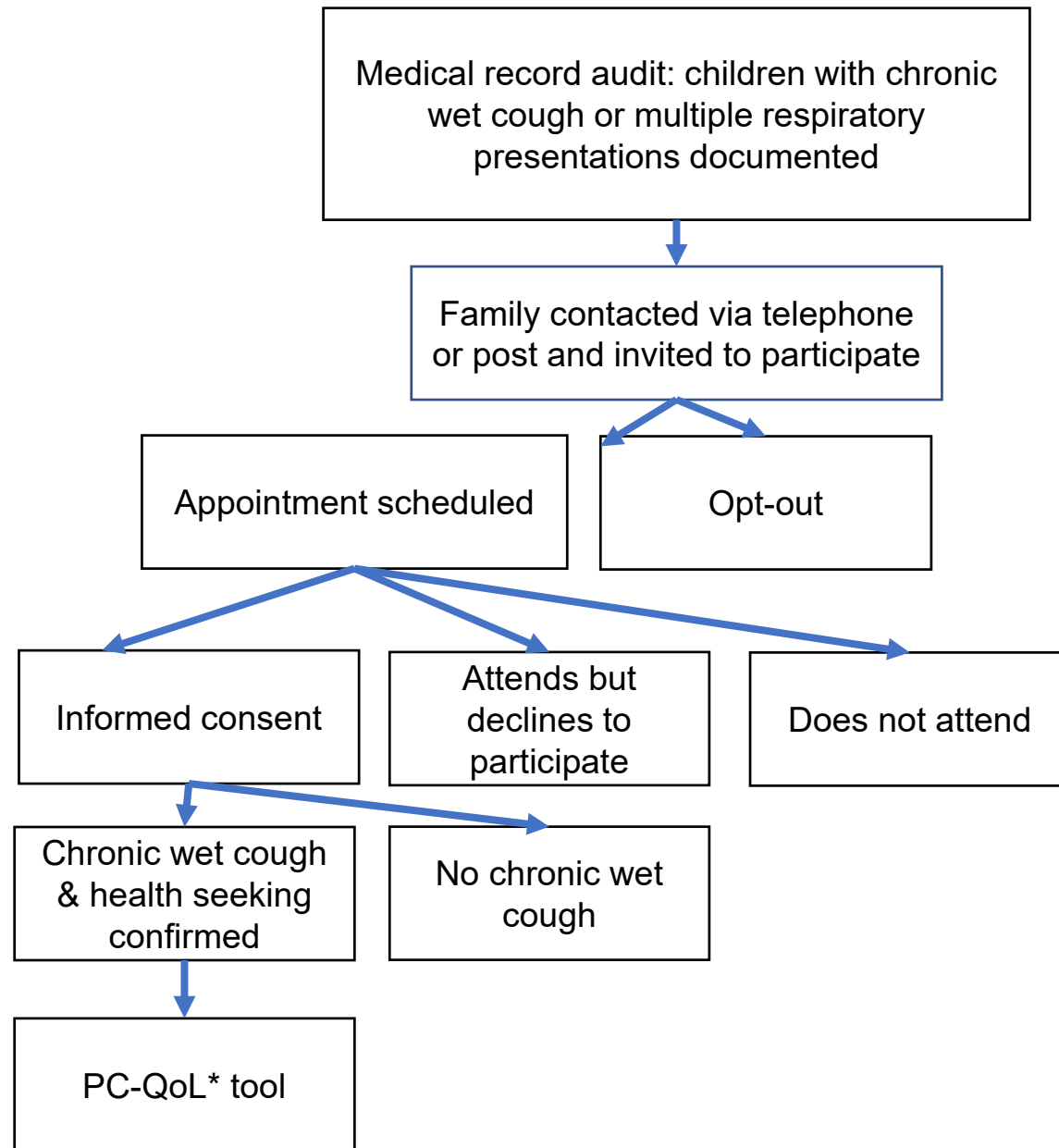

Supplement: Supplementary file 3 — Additional file 3. [file 12890_2022_2219_MOESM3_ESM.pdf]
